# Empagliflozin mitigates lipopolysaccharide-induced tracheal injury in rats via downregulation of IL-6/JAK/STAT3 signaling pathway and stem cell preservation: histological and molecular study

**DOI:** 10.1007/s00418-026-02531-4

**Published:** 2026-09-05

**Authors:** Nahla Reda Sarhan, Sally A. Elekhtiar, Rasha Elmowafy, Amira I. Shrief

**Affiliations:** 1 Medical Histology and Cell Biology Department, Faculty of Medicine, Mansoura University, Mansoura, Egypt; 2 Medical Histology and Cell Biology Department, Faculty of Medicine, Horus University–Egypt, New Damietta, Egypt; 3 Histology and Cell Biology Department, Faculty of Medicine, Kafrelsheikh University, Kafr El Sheikh, Egypt; 4 Medical Biochemistry and Molecular Biology Department, Faculty of Medicine, Mansoura University, Mansoura, Egypt; 5 Medical Biochemistry and Molecular Biology Department, Faculty of Medicine, Horus University–Egypt, New Damietta, Egypt

**Keywords:** Empagliflozin, Lipopolysaccharide, IL-6/JAK/STAT3 pathway, Epithelial–mesenchymal transition, Tracheal basal stem cells

## Abstract

Lipopolysaccharide (LPS) is a component of the Gram-negative bacterial cell wall and is considered a potent inflammatory inducer. Empagliflozin (EMPA) is a sodium-glucose co-transporter 2 inhibitor used in treatment of type 2 diabetes mellitus. Interleukin-6/Janus kinase/signal transducer and activator of transcription 3 (IL-6/JAK/STAT3) signaling pathway is essential in driving inflammation, fibrosis, and epithelial–mesenchymal transition (EMT). Tracheal basal stem cells are responsible for epithelial regeneration after injury. This study aimed to investigate the potential protective effects of EMPA against LPS-induced tracheal injury, with emphasis on EMT and tracheal basal stem cells. Sixty adult male rats were allocated into four groups: control; EMPA, which received EMPA (10 mg/kg/day) via oral gavage for 28 days; LPS, which received single intratracheal dose of LPS (5 mg/kg) on day 1; and LPS + EMPA, which received single LPS dose and EMPA for 28 days. Tracheal specimens were processed for biochemical, histological, immunohistochemical and RT-quantitative real-time PCR analysis. Compared to the LPS group, the LPS + EMPA group showed marked improvement of histological, ultrastructural ,and biochemical alterations including maintenance of epithelial and cilia integrity, decreased lipid peroxidation, stimulated antioxidant enzyme activities, reduced serum inflammatory cytokines, increased percentage area of E-cadherin (an epithelial marker) immunostaining, decreased percentage area of fibrosis, vimentin (a mesenchymal marker), and pro-apoptotic Bax; and enhanced percentage area of cytokeratin 5/6 (CK5/6) (stem cell marker). Moreover, EMPA downregulated the IL-6/JAK/STAT3 pathway involved in EMT. In conclusion, EMPA alleviated LPS-induced tracheal injury through its antioxidant and anti-inflammatory properties, downregulation of IL-6/JAK/STAT3 signaling pathway, and preservation of the tracheal basal stem cells.

## Introduction

The trachea has an essential role in maintaining airway patency and defense. However, it remains an underexplored target in inflammatory airway diseases. Research on tracheal injury is crucial, as structural and functional impairments in this organ can disrupt normal airflow, promote fibrosis, and contribute to chronic respiratory diseases, highlighting the need for targeted therapeutic interventions (Xie et al. 2021; Akparova et al. 2026).

Lipopolysaccharide (LPS), a major component of the Gram-negative bacterial cell wall, is considered a potent inducer of inflammation. LPS has been widely used to induce a model of airway injury and chronic respiratory diseases (Dianat et al. 2025). LPS binds to Toll-like receptor 4 (TLR4) on epithelial cells, activating the TLR4/nuclear factor-kappa B (NF-κB) signaling pathway, which leads to release of pro-inflammatory cytokines, including tumor necrosis factor alpha (TNF-α), interleukin-1β (IL-1β), and interleukin-6 (IL-6) (Wu et al. 2021). The upregulation of IL-6 triggers the IL-6/Janus kinase/signal transducer and activator of transcription 3 (IL-6/JAK/STAT3) signaling pathway. Activated Janus kinase 2 (JAK2) causes STAT3 phosphorylation, dimerization, and nuclear translocation which trigger transcription of genes involved in epithelial–mesenchymal transition (EMT), inflammation, and fibrosis, leading to tissue damage and airway remodeling (Kang et al. 2021; Wu et al. 2021).

EMT is a process in which the epithelial cells lose their polarity and adhesion properties and acquire mesenchymal characteristics that promote fibrosis and airway remodeling. EMT is characterized by decreased expression of epithelial markers such as E-cadherin and increased expression of mesenchymal markers such as α-smooth muscle actin (α-SMA) and vimentin (Mottais et al. 2023; Rout-Pitt et al. 2025). Immunohistochemical staining of E-cadherin and vimentin provides a reliable means to track the transition from epithelial to mesenchymal phenotype in LPS-induced tracheal injury (Dey et al. 2025).

The tracheal basal cells (BCs) function as stem cells. They maintain the integrity of the tracheal epithelium and regenerate it after injury. Tracheal basal stem cells are characterized by the expression of cytokeratin 5/6 (CK5/6), which serves as a reliable immunohistochemical marker for identifying this stem/progenitor compartment (Tadokoro et al. 2021; Liu et al. 2025a). Despite extensive research on LPS-induced lung injury, relatively few studies have specifically examined its impact on tracheal basal stem cells or its association with EMT within the tracheal epithelium (Han et al. 2026).

Empagliflozin (EMPA), a sodium-glucose co-transporter 2 (SGLT2) inhibitor, is used clinically in the treatment of type 2 diabetes mellitus (Abunada et al. 2026). By blocking glucose reabsorption in the renal tubules, it promotes glycosuria, thereby reducing plasma glucose levels. Beyond its glycemic effects, accumulating evidence indicates that EMPA exerts significant anti-inflammatory actions, including inhibition of NLRP3 inflammasome activation and subsequent reduction in pro-inflammatory cytokines such as TNFα, IL-6, IL-8, and IL-1β (Zhang et al. 2024). EMPA has been shown to attenuate inflammation and fibrosis in the heart, liver, and kidneys in experimental models of insulin resistance and diabetes mellitus (Maayah et al. 2021).

The potential role of EMPA in modulating the LPS-induced tracheal injury remains insufficiently investigated. Moreover, the involvement of specific signaling pathways, particularly the IL-6/JAK/STAT3 axis and its effect on tracheal basal stem cells, have not been clearly elucidated. Therefore, the current work aims to provide novel insights into the potential protective effects of EMPA against LPS-induced tracheal injury, with particular emphasis on its role in modulating EMT and tracheal basal stem cells using biochemical, histological, immunohistochemical, and molecular analyses.

## Materials and methods

### Chemicals

Lipopolysaccharide (LPS), in the form of powder dissolved in sterile saline (0.9% NaCl), was purchased from Almolok Chemicals, Kafr El-Sheikh, Egypt (a product of Solarbio, Cat. No. L8880).

Carboxymethyl cellulose sodium (CMC) was purchased from Almolok Chemicals, Kafr El-Sheikh, Egypt (a product of CP Kelco company (CEKOL 30000) (CAS # 9004-32-4). It was available in the form of solid odorless white powder in a 250-g container. A 0.5% CMC solution was prepared by adding 0.5 g of CMC to 100 mL of distilled water and stirring continuously for at least 2 h or overnight to avoid clumping and to ensure complete hydration and uniform viscosity (Hashem et al. 2025).

Empagliflozin (EMPA), a product of Boehringer Ingelheim, Germany (trade name Jardiance®), was purchased from a local pharmacy. It was available in the form of 10-mg tablets that were suspended in 0.5% CMC solution. To prepare an EMPA suspension with a concentration of 1 mg/mL, 10 mg of EMPA was added to 10 mL of 0.5% CMC solution (Chung et al. 2023). The suspension was stored at 4 °C and shaken well before each use to ensure uniform dispersion.

### Study protocol and experimental animals

The study protocol approved by the Institutional Animal Care and Use Committee (KFS-IACUC), Faculty of Medicine, Kafrelsheikh University (approval number KFS-IACUC/275/2025). All animal experiments were conducted in accordance with the ethical guidelines of KFS-IACUC. The study was carried out at the animal house of the Faculty of Pharmacy, Kafrelsheikh University. Sixty adult male Sprague–Dawley rats with a weight between 180 and 200 g were used. The rats were housed under standard laboratory conditions in separate cages and were kept at room temperature (25 ± 2 °C), humidity, and 12-h light–dark cycles. The animals were fed a standard chow diet and allowed free access to water. The experimental duration was 28 days.

After 1 week of accommodation, the rats were randomly assigned into four groups using the online GraphPad random number calculator (https://www.graphpad.com/quickcalcs/randomize1/):

Control group (*n* = 24 rats), was subdivided into two subgroups: control areceived a single dose of sterile saline (0.9% NaCl) via intratracheal instillation; control breceived CMC 0.5%, 0.5 mL/day) via oral gavage for 28 days.

EMPA group (*n* = 12 rats), received empagliflozin (10 mg/kg/day) dissolved in 0.5% CMC via oral gavage for 28 days. This dose was selected on the basis of its demonstrated antioxidant, anti-inflammatory, and antiapoptotic effects in experimental models (Asgari and Kalhori 2025).

LPS group (*n* = 12 rats), received a single dose of LPS (5 mg/kg) dissolved in sterile saline intratracheally on day 1 of the experiment. This dose was selected to establish a reproducible model of tracheal injury (Xie et al. 2021).

LPS + EMPA group (*n* = 12 rats), was treated as LPS group, followed by empagliflozin (10 mg/kg/day) via oral gavage for 28 days.

### Obtaining and processing of specimens

At the end of the experiment, the rats were anesthetized by intraperitoneal injection of sodium pentobarbital (40 mg/kg) (Dutton 3rd et al. 2019). Blood samples were then collected from the retro-orbital venous plexus of all groups. Six rats from each group were subjected to intracardiac perfusion by 2.5% glutaraldehyde with 0.1 M phosphate buffer at pH 7.4 for electron microscopic examination. This was done to partially fix the specimens and prevent the fixative from affecting the quality and outcomes of the other experiments. Tracheal specimens were dissected out and processed for biochemical, histological, and molecular studies. To minimize subjective bias, blinding was employed at both the data collection and processing stages. The investigators were blinded to the treatment allocations, and the subsequent data analysis was performed by an analyst who was unaware of the group key until the analysis was completed.

### Biochemical study

#### Preparation of tracheal tissue homogenates for estimation of oxidative stress markers

Samples from the trachea of all groups (6 rats each) were washed with a phosphate-buffered saline (pH 7.4) solution containing heparin to remove RBCs and clots. After homogenizing the tissue specimens in 8 mL of cold buffer (including 50 mM potassium phosphate and 1 mM EDTA) per gram tissue, the samples were centrifuged at 1500 × *g* for 15 min at 4 °C. The liquid supernatant was then collected and stored at − 80 °C for later use.

#### Assessment of malondialdehyde (MDA) content and activity of antioxidant enzymes

Tissue content of the lipid peroxidation marker (MDA) (in nmol/gm tissue) and activities of catalase (CAT) and superoxide dismutase (SOD) (U/g tissue) were measured using commercial colorimetric kits according to the manufacturer’s instructions (Bio-Diagnostics, Dokki, Giza, Egypt (Cat. No. MD 2529, CA 2517 and SD 2521, respectively).

#### Measurement of serum proinflammatory cytokines

Following separation of serum from the collected blood samples, TNF-α, IL-1β, and IL-6 were measured in serum samples using a sandwich ELISA technique and expressed as pg/mL. TNF-α and IL-1β were measured according to the manufacturer’s instructions using ELISA kits purchased from CUSABIO, USA (Cat. Nos. CSB E11987r and CSB-E08055r, respectively). IL-6 was measured using Rat IL-6 Immunoassay Kit purchased from R&D Systems, USA (Cat. No. R6000B).

### Histological study

#### Light microscopy study

Specimens from trachea were fixed in 10% neutral buffered formalin for 24 h at room temperature, dehydrated in ascending alcohol grades, cleared in xylene, and then embedded in paraffin wax. Sections of 5–6 μm thickness were cut and used for hematoxylin and eosin (H&E) staining for routine histological examination (Bancroft and Layton 2019a); Masson’s trichrome staining (Bancroft and Layton 2019b) for demonstration of collagenous fibers and estimation of fibrosis; and immunohistochemical study.

#### Immunohistochemical study

The streptavidin–biotin complex technique (Sanderson et al. 2018) was used to immunolocalize the E-cadherin as an epithelial marker, vimentin as a mesenchymal tissue marker (Dey et al. 2025), Bcl2-associated-X protein (Bax) for apoptosis (Liu et al. 2025b), and CK5/6 as a marker for tracheal basal stem cells (Cabibi et al. 2020). In brief, following rehydration of the deparaffinized sections, they were incubated in citrate buffer (pH 6) for 22 min in the microwave to retrieve the antigen and then washed with cold water. Endogenous peroxidase activity was inhibited by incubating the sections in distilled water containing 3% H_2_O_2_. To block the nonspecific background staining, the sections were subsequently incubated with normal goat serum (XO9O7; Dako, Carpinteria, California, USA). The sections were then incubated with the following primary antibodies: anti-E cadherin (rabbit polyclonal antibody, Cat. No. GB11082, dilution 1:500, Servicebio), anti-vimentin (mouse monoclonal antibody, Cat. No. MS-129-R7, diluted 1:100, Thermo Fisher Scientific, Runcorn, Cheshire, UK), anti-Bax (rabbit polyclonal antibody, Cat. No. bs-0127R, diluted 1:200, Bioss, Beijing, China), and anti-CK5/6 (rabbit polyclonal antibody, Cat. No. PA5- 116450 , diluted 1:50, Invitrogen, Thermo Fisher Scientific, USA) for 60 min. Tris-buffered saline (TBS) was used for slide washing and then sections were then incubated with a biotinylated universal secondary antibody (goat anti-mouse immunoglobulin). The slides were then incubated with streptavidin–biotin–peroxidase complex solution (DAKO-Universal LSAB + System-HRP Kit-K0690; Dako, Carpinteria, CA, USA) for 30 min. The immune reaction was visualized using 3,3′-diaminobenzidine (DAB), which was subsequently counterstained with hematoxylin. After omitting the primary antibody, the same procedures were used to prepare negative control slides. The specificity of the primary antibodies was validated according to the manufacturer’s datasheets that provided specific immunohistochemical staining in the positive controls as follows: rat colon for E-cadherin (available at: https://www.servicebio.com/goodsdetail?id=148834), sarcoma for vimentin (available at: https://share.google/7mzGOfE9hC31WvIUS), rat brain for Bax (available at: https://www.biossantibodies.com/products/datasheets/bs-0127R), and mouse skin for CK5/6 (available at: https://www.thermofisher.com/antibody/product/Cytokeratin-5-6-Antibody-Polyclonal/PA5-116450). Brown membranous or cytoplasmic reaction was regarded as a positive reaction for E-cadherin and brown cytoplasmic reaction was considered positive for vimentin, Bax, and CK5/6.

#### Electron microscopy study

Small pieces from the trachea measuring 1 mm were fixed for 2 h in a solution of 2.5% glutaraldehyde with 1 M sodium cacodylate buffer. The tracheal specimens were then post-fixed in 1% osmium tetroxide, dehydrated in ascending alcohol grades, immersed in propylene oxide, and embedded in epoxy resin; thereafter, 1-µm semi-thin sections were obtained and underwent staining with 1% toluidine blue ready for examination by light microscopy. Ultrathin sections (60–80 nm thick) were cut, stained with uranyl acetate and lead citrate (Woods and Stirling 2018), and examined using a JEOL-JEM-1400 plus transmission electron microscope (TEM) at The Electron Microscopy Unit, Faculty of Science, Alexandria University, Egypt.

### Quantification of mRNA by reverse transcriptase quantitative real-time polymerase chain reaction (RT-qPCR)

After homogenization of tracheal tissue samples from each animal (6 rats/group) in liquid nitrogen, the total RNA was extracted using the QIAzol reagent (Qiagen, Germany, Cat. No. 79306) according to the manufacturer's recommendations. Thermo Scientific Nano Drop One (USA) was used to assess the quantity and purity of RNA yield of each sample. Subsequently, 1 μg of RNA was converted into cDNA using the Revertaid First Strand cDNA Synthesis Kit (Thermoscientific, USA, Cat. No. K1621) and a Proflex Thermal Cycler (Applied Biosystems, USA). RT-qPCR technique was applied to amplify the cDNA templates by using the following reaction conditions: 10 μL of HERA ^plus^ SYBR green PCR Master Mix (Willowfort, UK, Cat. No. WF10308001), 2 μL of cDNA template, 2 μL of forward and reverse gene primer with a concentration of 10 pmol/μL, and 6 μL of nuclease-free water. The used device was real-time PCR device (Azure Cielo 6, USA), and the used program was started with initial denaturation at 95 °C for 2 min, followed by 40 cycles of annealing and extension at 60 °C for 30 s. The employed primer pair sequences (Vivantis, Malaysia) are shown in Table 1. The specific primers of the genes were designated using the Primer3 Plus software (https://www.bioinformatics.nl/cgi-bin/primer3plus/primer3plus.cgi) and their specificity was checked by the Primer-BLAST program (https://www.ncbi.nlm.nih.gov/tools/primer-blast/). To normalize RNA expression levels, the glyceraldehyde-3-phosphate dehydrogenase gene (*GAPDH*) was utilized as an internal control gene. After confirmation of the specificity of the PCR results by analysis of melting curves, the relative gene expression levels were expressed using the following equation: ΔCt = Ct target gene − Ct housekeeping gene. The fold change in gene expression was then calculated using the 2^−ΔΔCT^ method (Livak and Schmittgen 2001).

**Table 1:** Sequences of genes’ specific primers used in RT-qPCR

Gene	Sequence	Product size	RefSeq
*IL-6*	Forward: TCCTACCCCAACTTCCAATGCTC	79 bp	NM_012589.2
	Reverse: TTGGATGGTCTTGGTCCTTAGCC		
*JAK2*	Forward: GACGGGAAGGTCTTGGAAATAG	284 bp	NM_031514.1
	Reverse: TCAACGGCAAAGGTCAGGAA		
*STAT3*	Forward: AACTGCTTGCCTTGACCAC	129 bp	NM_001430046.1
	Reverse: CGCCTTGCCTTCCTAAATAC		
*GAPDH*	Forward: TGGGAAGCTGGTCATCAAC	78 bp	NM_017008.4
	Reverse: GCATCACCCCATTTGATGTT		

### Image acquisition and computer-aided digital image analysis

For histomorphometric analysis, photos from the slides were captured by Leica ICC50 W color digital camera (5-megapixel; ICC50W2338, Germany) attached to a Leica microscope (DM500; Germany) using ×40 high-power objective lens (×400 magnification) with numerical aperture (NA) = 0.65. Six tracheal sections stained with H&E and Masson’s trichrome, and immunohistochemical markers were examined from each group (6 rats/group). In each section, six randomly selected non-overlapping fields were quantitatively analyzed. Epithelial thickness was measured perpendicular to the basement membrane at six different points per field. In addition, the percentage area (%) of collagenous fibers, E-cadherin, vimentin, Bax, and CK5/6 immunostaining was assessed. For digital image analysis, VideoTesT Morphology software program (Russia, Saint Petersburg) was used for specific built-in routine for automated object analysis.

### Statistical analysis

Data was analyzed using Statistical Package for Social Science software (SPSS) software version 26 (SPSS Inc., PASW statistics for windows version 26. Chicago, USA, SPSS Inc.). Normality of data distribution was assessed using the Shapiro–Wilk test and all datasets were normally distributed. The data was described as mean ± standard deviation (SD). For normally distributed data one-way analysis of variance (ANOVA) test was used to compare more than two independent groups followed by post hoc Tukey test to detect pairwise comparison. Multiple-comparison adjustments were applied consistently across all datasets. Statistical significance was considered at *p* value < 0.05 (Zhang 2022).

## Results

The control subgroups (a and b) and EMPA group demonstrated similarity in the structural and ultrastructural features, and in the immunohistochemical staining pattern. Therefore, control group photos are presented in the current study to avoid repetition of similar results with the EMPA group. Moreover, these groups were statistically comparable to each other in the estimated biochemical and histomorphometric parameters; and in the relative expression of genes quantified by RT-qPCR.

### EMPA mitigated lipid peroxidation and enhanced activity of antioxidant enzymes in trachea of LPS-treated rats

**Fig. 1:**
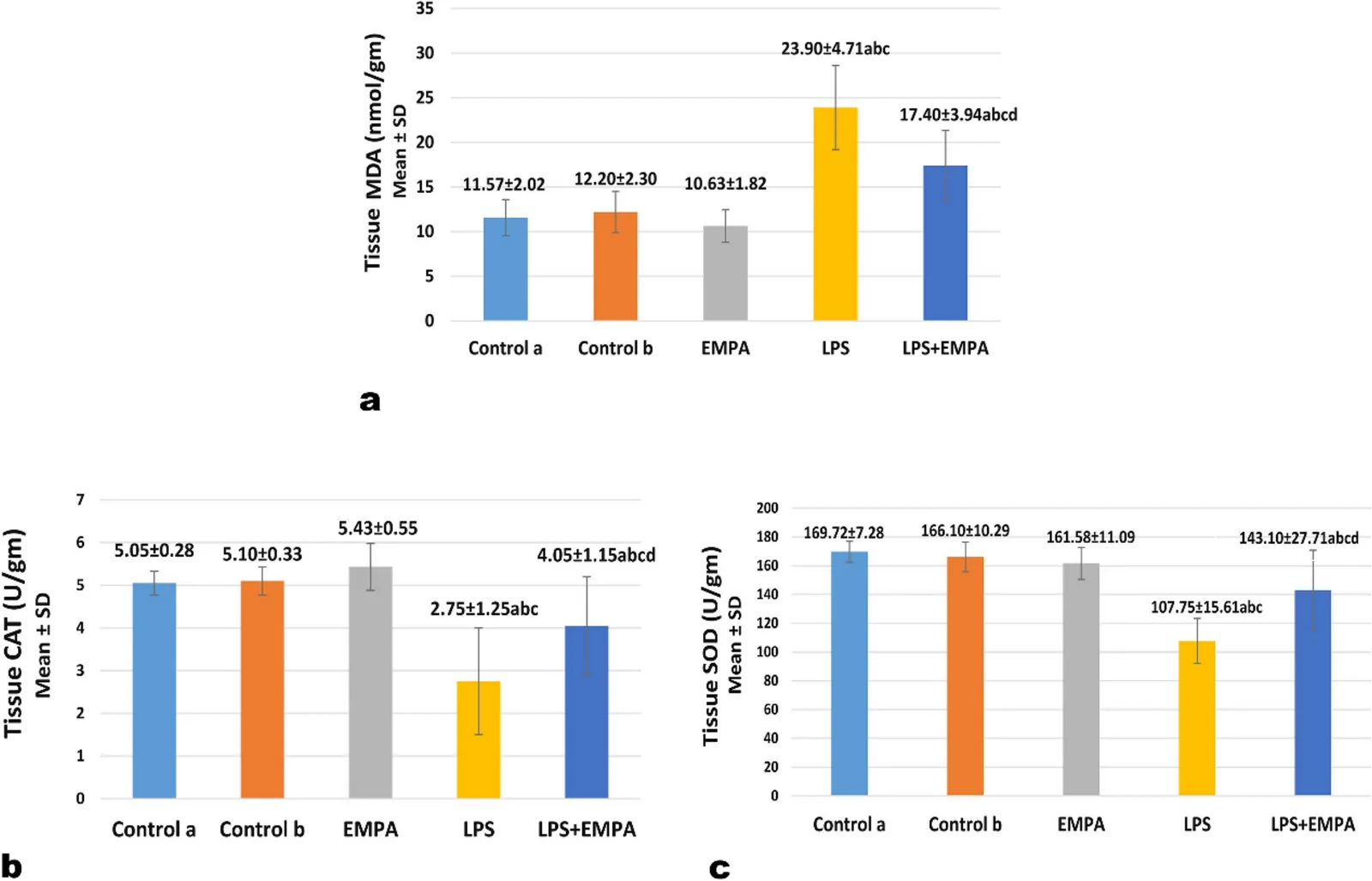
Tissue MDA content (nmol/gm) (**a**), CAT (**b**), and SOD (**c**) enzyme activities (nmol/gm) in the trachea of the study groups. One-way ANOVA test is used. Data are expressed as mean ± SD. *P* < 0.05 is significant; a indicates significance against control group a, b significance against control group b, c significance against EMPA group, d significance against LPS group. (*n* = 6 rats/group)

Tracheal MDA content significantly increased (*p* < 0.05) in the LPS group compared to both control and EMPA groups. On the other hand, it showed a significant decrease (*p* < 0.05) in the LPS + EMPA group compared to the LPS group; however, it remained significantly higher (*p* < 0.05) than in the control and EMPA groups (Fig. 1a). The activities of tissue CAT and SOD enzymes were significantly lower (*p* < 0.05) in the LPS and LPS + EMPA groups relative to the control and EMPA groups. But the activity of both enzymes was significantly higher (*p* < 0.05) in the LPS + EMPA group compared to the LPS group (Fig. 1b, c).

### EMPA mitigated increase in serum level of pro-inflammatory cytokines in LPS-treated rats

**Fig. 2:**
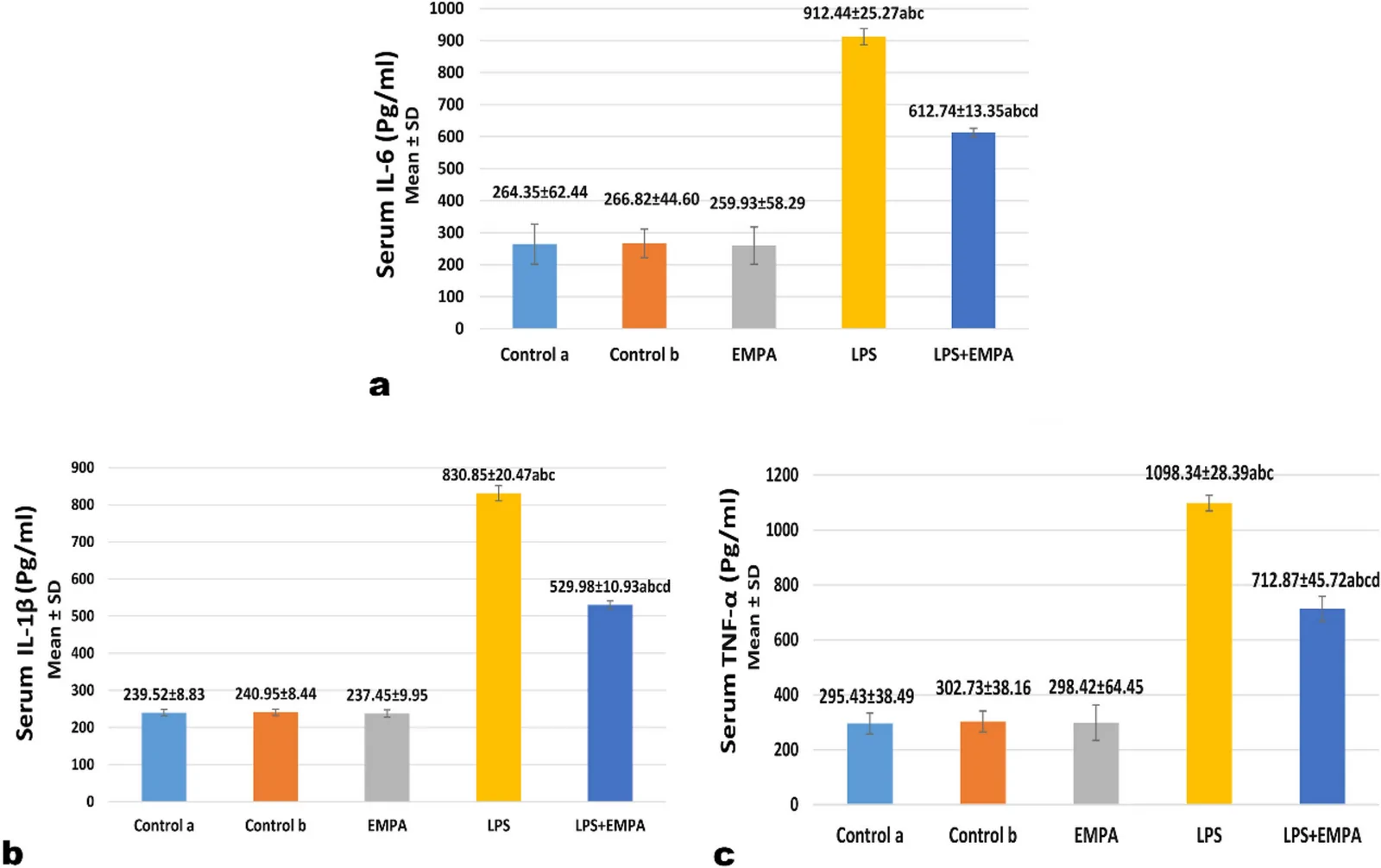
Serum level of IL-6 (**a**), IL-1β (**b**), and TNF-α (**c**) in the study groups. One-way ANOVA test is used. Data are expressed as mean ± SD. *P* < 0.05 is significant; a indicates significance against control group a, b significance against control group b, c significance against EMPA group, d significance against LPS group. (*n* = 12 rats/group)

The serum levels of IL-6, IL-1β, and TNF-α cytokines were significantly higher (*p* < 0.05) in the LPS and LPS + EMPA groups relative to the control and EMPA groups. However, these levels significantly reduced (*p* < 0.05) in the LPS + EMPA group compared to the LPS group (Fig. 2).

### EMPA mitigated tracheal histological alterations in LPS-treated rats

**Fig. 3:**
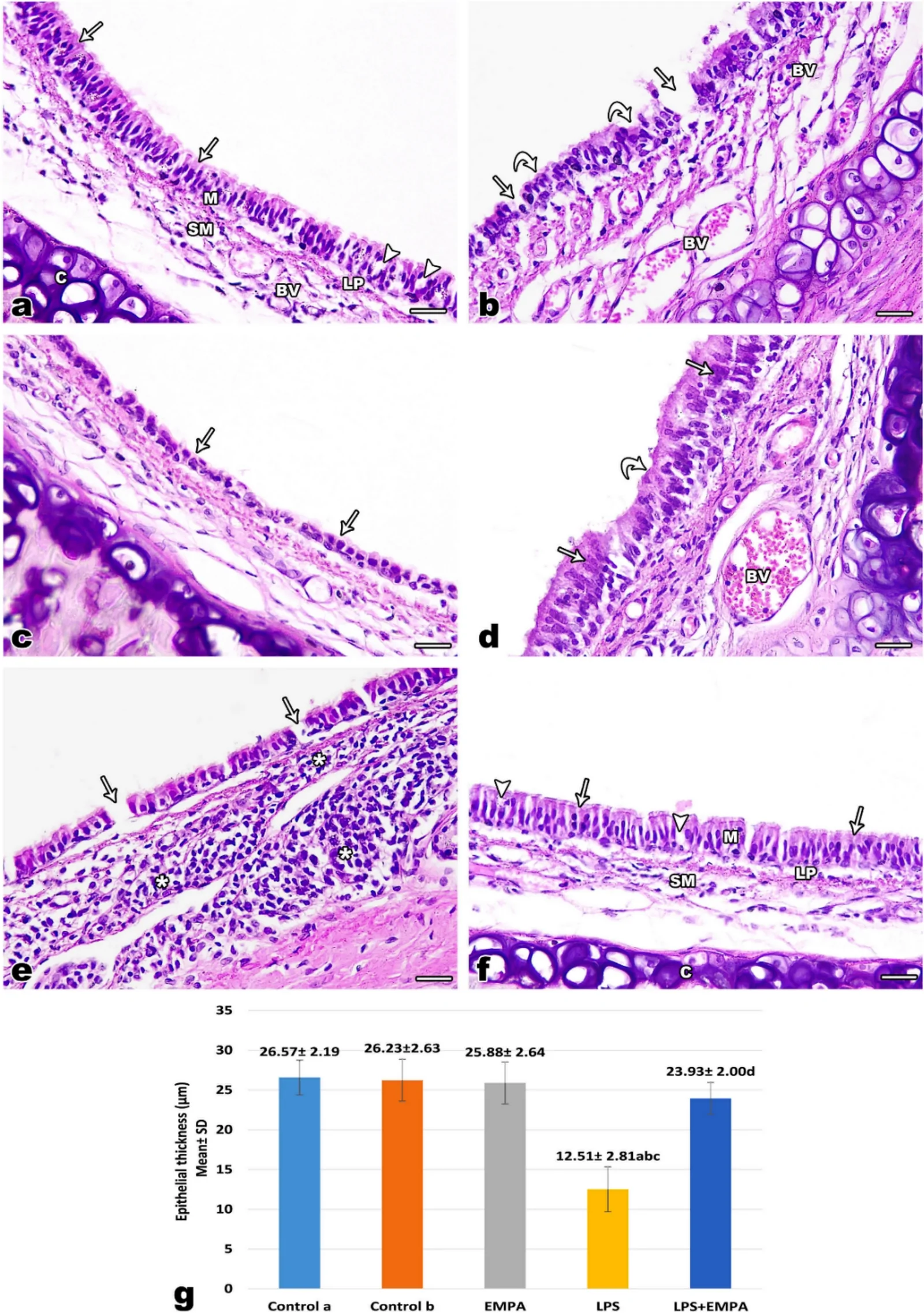
H&E-stained tracheal sections from the study groups (**a** control group; **b**–**e** LPS group;** f** LPS + EMPA group). **a** Part of the tracheal wall shows mucosa (M), submucosa (SM), and hyaline cartilage (c). The mucosa is formed of pseudostratified columnar ciliated epithelium (arrows) with goblet cells (arrowheads) and underlying lamina propria (LP). Blood vessels (BV) are seen in the lamina propria and submucosa. **b** Disorganized tracheal epithelium with almost complete loss (arrows) in some areas. Most ciliated cells show disarranged or lost cilia (curved arrows). Dilated congested blood vessels (BV) are observed. **c** shows  an area of mucosa lined with a single layer of cuboidal epithelial cells (arrows) with complete loss of cilia. **d** Part of the mucosa shows epithelial irregularity and hyperplasia (arrows). Some epithelial cells exhibit loss of their cilia (curved arrow). Dilated congested blood vessels (BV) are observed. **e** Lost epithelial cells (arrows) and heavy inflammatory cell infiltration (asterisks) in the lamina propria and submucosa are observed. **f** The tracheal mucosa (M) is relatively preserved and showed pseudostratified columnar ciliated epithelium (arrows) with goblet cells (arrowheads) and the underlying lamina propria (LP). Notice the submucosa (SM) and part of hyaline cartilage (c) (×400; scale bar = 25 μm). **g** Epithelial thickness (µm) of the trachea in the study groups. One-way ANOVA test is used. Data are expressed as mean ± SD. *P* < 0.05 is significant; a indicates significance against control group a, b significance against control group b, c significance against EMPA group, d significance against LPS group. (*n* = 6 rats/group)

H&E-stained sections of the control and EMPA groups showed normal histology of the tracheal wall, which comprised mucosa, submucosa, plates of hyaline cartilage, and adventitia. Here we present the results for the mucosa and submucosa because the majority of the histological changes were detected in these two layers. The mucosa comprised pseudostratified columnar ciliated epithelium with intervening goblet cells and an underlying lamina propria. Blood vessels were present in the connective tissue of the lamina propria and submucosa (Fig. 3a). In the LPS group, the tracheal mucosa demonstrated a variety of remarkable histological changes: it appeared disorganized with areas of complete loss of epithelial cells (denudation) and the majority of the ciliated cells showed disorganized or lost cilia. Other mucosal areas were lined with a single layer of cuboidal epithelial cells with complete loss of cilia. In addition, there was epithelial hyperplasia and irregularity in some mucosal areas. Heavy inflammatory cell infiltration in the lamina propria and submucosa and dilated congested submucosal blood vessels were noted (Fig. 3b–e). In contrast, the LPS + EMPA group showed marked improvement compared to the LPS group. The trachea exhibited a preserved architecture. Most of the ciliated epithelial cells showed well-organized cilia (Fig. 3f). The epithelial thickness was significantly lower (*p* < 0.05) in the LPS group compared to both control and EMPA groups. It was significantly elevated (*p* < 0.05) the LPS + EMPA group compared to the LPS group, but non-significantly different from the control group (Fig. 3g).

### EMPA mitigated tracheal fibrosis in LPS-treated rats

**Fig. 4:**
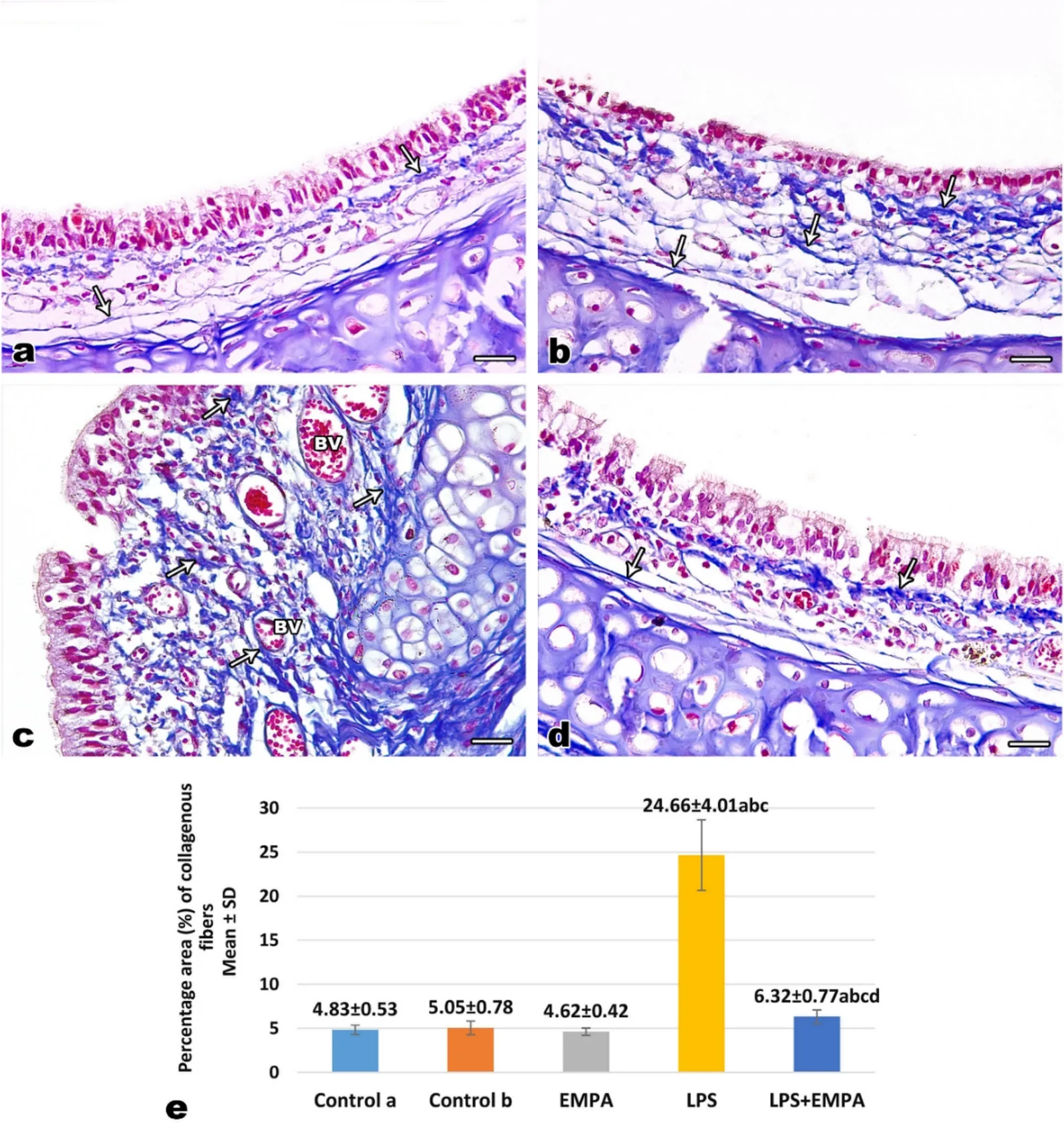
**a**–**d** Masson’s trichrome-stained tracheal sections from the study groups (**a** control group;** b**,** c** LPS group;** d** LPS + EMPA group). **a** showing  collagenous fibers (arrows) in the lamina propria and submucosa. **b** Area of denuded epithelium with excess amounts of collagenous fibers (arrows) in the underlying lamina propria and submucosa. **c **Area of hyperplastic epithelium showing extensive deposition of collagenous fibers (arrows) in the lamina propria, submucosa, and around congested blood vessels (BV). **d**  showing  moderate amount of collagenous fibers (arrows) in the lamina propria and submucosa (×400; scale bar = 25 μm). **e** Percentage area (%) of collagenous fibers in the trachea of study groups. One-way ANOVA test is used. Data are expressed as mean ± SD. *P* < 0.05 is significant; a indicates significance against control group a, b significance against control group b, c significance against EMPA group, d significance against LPS group. (*n* = 6 rats/group)

Masson’s trichrome-stained sections of the control and EMPA groups revealed some collagenous fibers in the lamina propria and submucosa of the trachea. The LPS group showed excessive collagenous fibers in the lamina propria, submucosa, and around the congested blood vessels. On the other hand, the LPS + EMPA group showed moderate amounts of collagenous fibers in the lamina propria and submucosa (Fig. 4a–d). The percentage area (%) of collagenous fibers was significantly higher (*p* < 0.05) in the LPS group relative to control and EMPA groups. It was significantly lower (*p* < 0.05) in the LPS + EMPA group than the LPS group but still significantly higher (*p* < 0.05) relative to both control and EMPA groups (Fig. 4e).

### EMPA enhanced immune expression of tracheal E-cadherin in LPS-treated rats

**Fig. 5:**
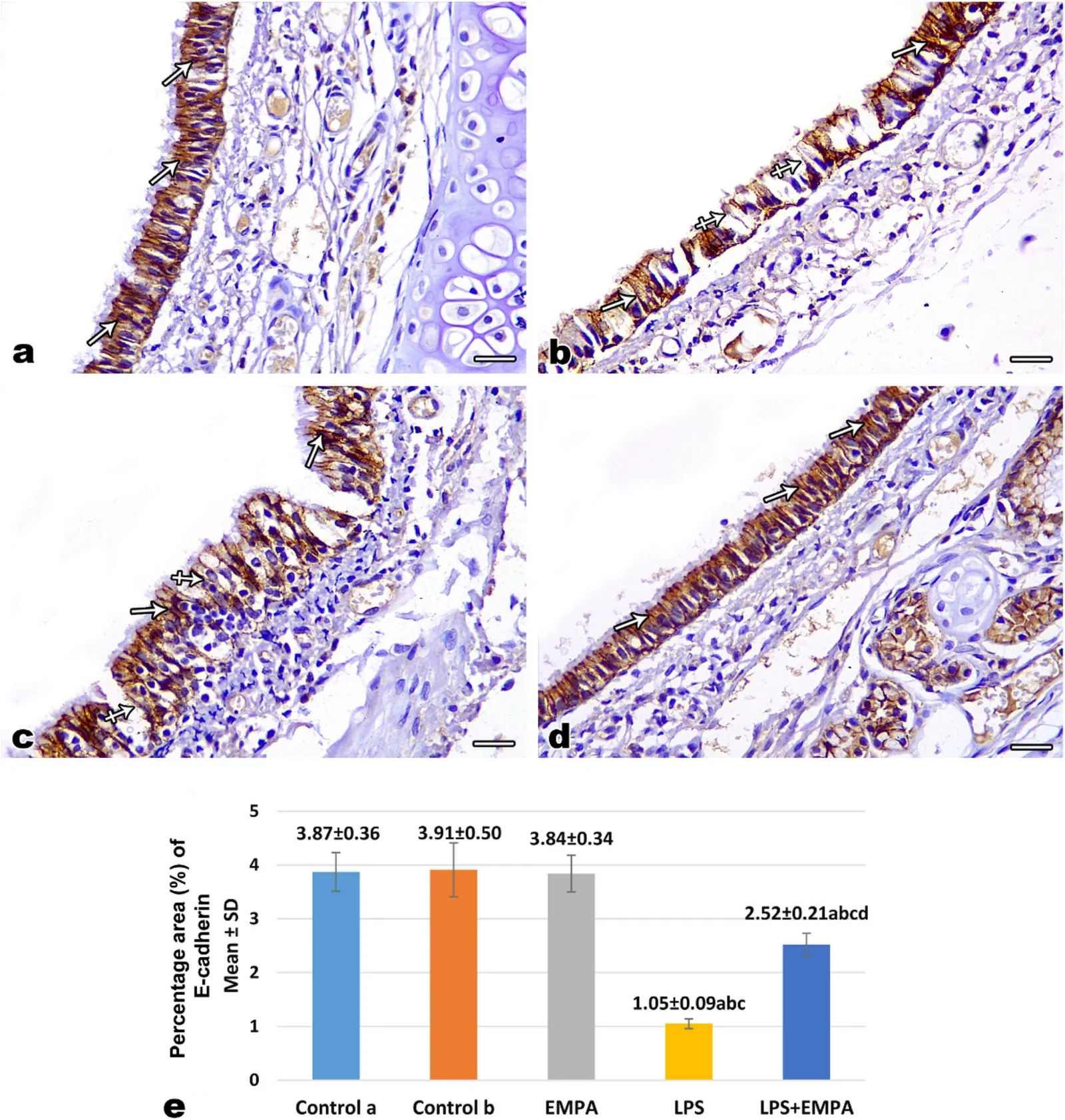
**a**–**d** E-cadherin immunostaining in the trachea of the studied groups (**a** control group;** b**,** c** LPS group;** d** LPS + EMPA group). **a** showing  strong membranous E-cadherin immunostaining (arrows) in the epithelial cells. **b**,** c** showing  moderate membranous E-cadherin immunostaining (arrows) in the epithelial lining with areas of focal loss of membrane staining (crossed arrows). **d** showing  strong membranous E-cadherin immunostaining (arrows) in the epithelial cells (anti-E-cadherin ×400, scale bar = 25 μm). **e** Percentage area (%) of tracheal E-cadherin immunostaining of study groups. One-way ANOVA test is used. Data are expressed as mean ± SD. *P* < 0.05 is significant; a indicates significance against control group a, b significance against control group b, c significance against EMPA group, d significance against LPS group. (*n* = 6 rats/group)

The control and EMPA groups showed strong membranous E-cadherin immune expression in the tracheal epithelial cells. The LPS group demonstrated weaker membranous E-cadherin immune expression with regions of focal loss of membrane immunostaining. In contrast, the LPS + EMPA group showed enhanced strong membranous E-cadherin immune expression in the epithelial cells (Fig. 5a–d). The percentage area (%) of E-cadherin immunostaining showed a significant reduction (*p* < 0.05) in the LPS group compared to the control and EMPA groups. It showed a significant elevation (*p* < 0.05) in the LPS + EMPA group compared to the LPS group and a significant reduction (*p* < 0.05) compared to the control and EMPA groups (Fig. 5e).

### EMPA mitigated EMT in LPS-treated rats through decreased tracheal vimentin immune expression

**Fig. 6:**
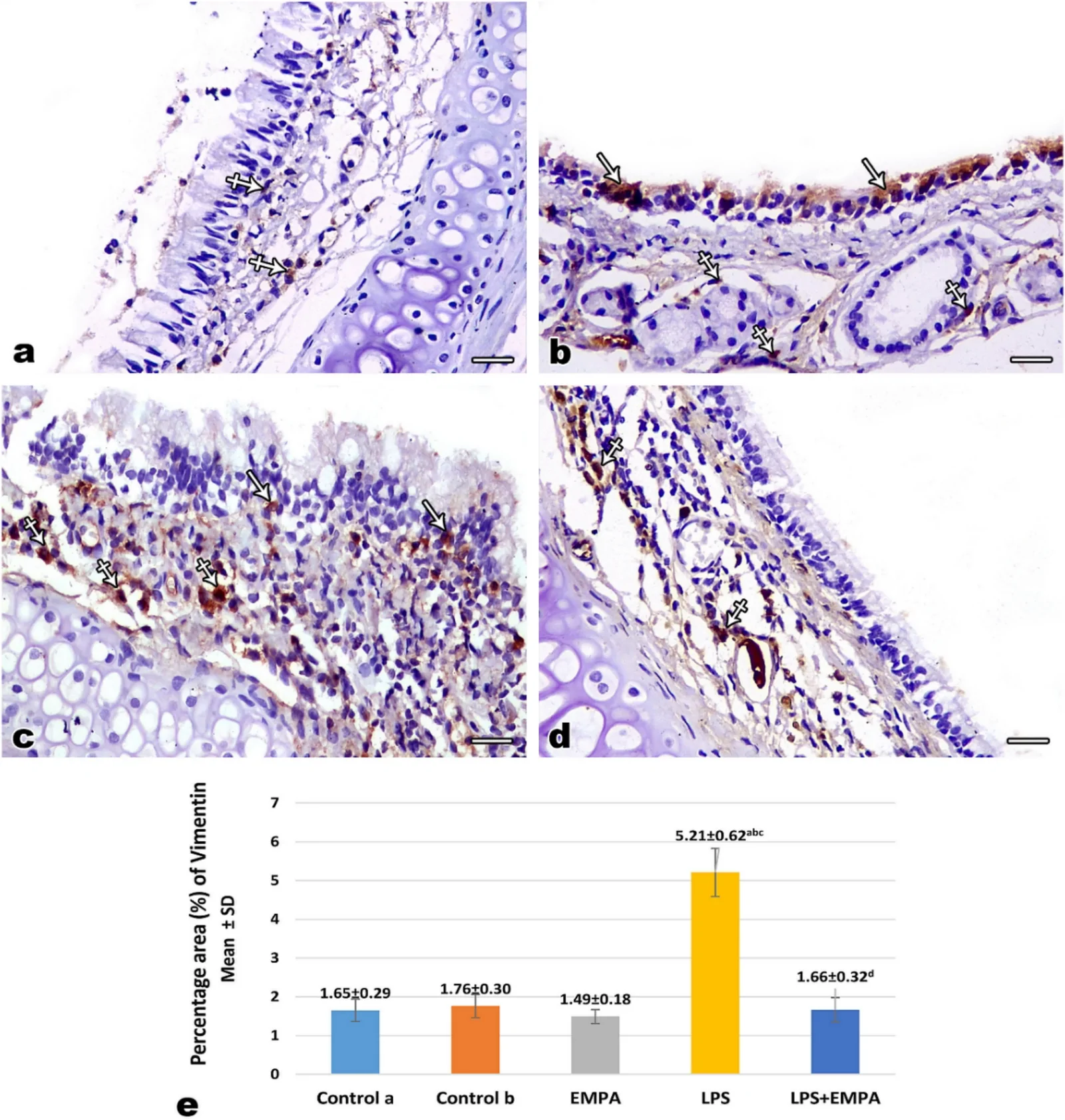
**a**–**d** Vimentin immunostaining in the trachea of the study groups (**a** control group;** b**,** c** LPS group;** d** LPS + EMPA group). **a** showing  mild cytoplasmic vimentin immune reaction in the connective tissue cells of lamina propria and submucosa (crossed arrows). Negative immunostaining is observed in the epithelial cells. **b**,**c** showing  strong cytoplasmic vimentin immunostaining in the epithelial cells (arrows) and in the connective tissue cells of lamina propria and submucosa (crossed arrows). **d** showing  moderate vimentin immunostaining in the connective tissue cells of lamina propria and submucosa (crossed arrows) with apparently negative immunostaining in the epithelial cells (anti-vimentin ×400; scale bar = 25 μm). **e** Percentage area (%) of tracheal vimentin immunostaining of study groups. One-way ANOVA test is used. Data are expressed as mean ± SD. *P* < 0.05 is significant; a indicates significance against control group a, b significance against control group b, c significance against EMPA group, d significance against LPS group. (*n* = 6 rats/group)

Mild cytoplasmic vimentin immunostaining was found in the connective tissue cells of the lamina propria and submucosa of the control and EMPA groups with negative reaction in the tracheal epithelium. In the LPS group strong vimentin immune reaction appeared in the tracheal epithelial cells together with the lamina propria and submucosal connective tissue cells. Whereas the LPS + EMPA group revealed negative vimentin immune reaction in the epithelial cells and moderate reaction in the lamina propria and submucosal connective tissue cells (Fig. 6a–d). The percentage area (%) of vimentin immunostaining was significantly higher (*p* < 0.05) in the LPS group compared to the control and EMPA groups. However, it was significantly lower (*p* < 0.05) in the LPS + EMPA group compared to the LPS group with no significant difference compared to both control and EMPA groups (Fig. 6e).

### EMPA mitigated apoptosis in LPS-treated rats through decreased tracheal Bax immune expression

**Fig. 7:**
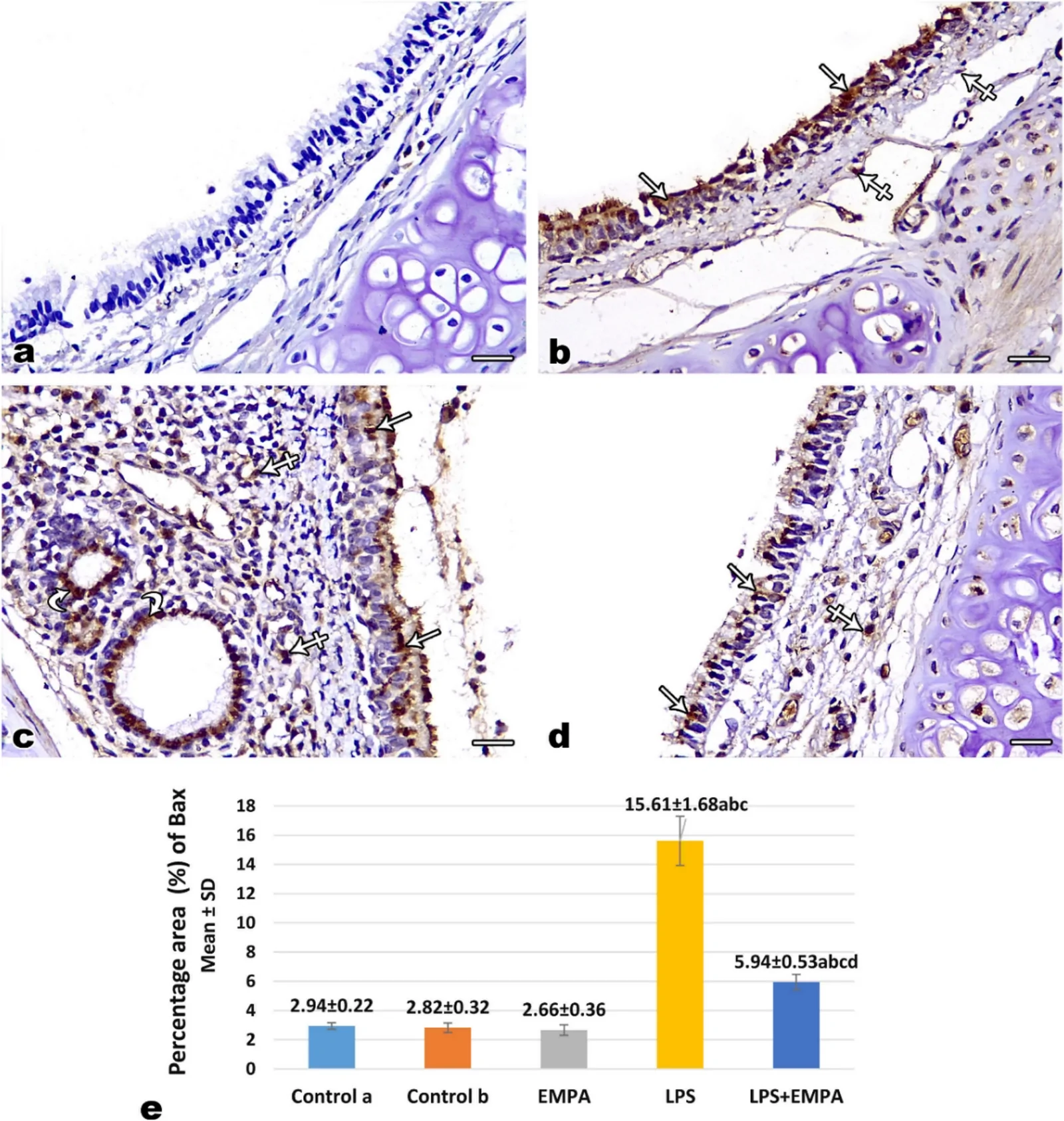
**a**–**d** Bax immunostaining in the trachea of the studied groups (**a** control group;** b**,** c** LPS group;** d** LPS + EMPA group). **a** showing  apparently negative Bax immunostaining. **b**, **c** showing  strong cytoplasmic Bax immunostaining in most cells of the epithelium (arrows), lamina propria, and submucosa (crossed arrows) and in submucosal glands (curved arrows). **d** showing  mild cytoplasmic immunostaining in some epithelial (arrows), lamina propria, and submucosal cells (crossed arrows) (anti-Bax, ×400; scale bar = 25 μm). **e** Percentage area (%) of tracheal Bax immunostaining of study groups. One-way ANOVA test is used. Data are expressed as mean ± SD. *P* < 0.05 is significant; a indicates significance against control group a, b significance against control group b, c significance against EMPA group, d significance against LPS group. (*n* = 6 rats/group)

Negative Bax immune expression was found in the control and EMPA groups. In the LPS group a strong cytoplasmic Bax immune expression was found in most tracheal epithelial cells and in the connective tissue cells of lamina propria and submucosa. On the other hand, in the LPS + EMPA group there was mild Bax immune expression in some cells of the epithelium, lamina propria, and submucosa (Fig. 7a–d). Percentage area (%) of Bax immunostaining showed a significant increase (*p* < 0.05) in the LPS and LPS + EMPA groups compared to the control and EMPA groups. However, it exhibited a significant reduction (*p* < 0.05) in the LPS + EMPA group compared to the LPS group (Fig. 7e).

### EMPA preserved tracheal basal stem cells in LPS-treated rats through enhanced CK5/6 immune expression

**Fig. 8:**
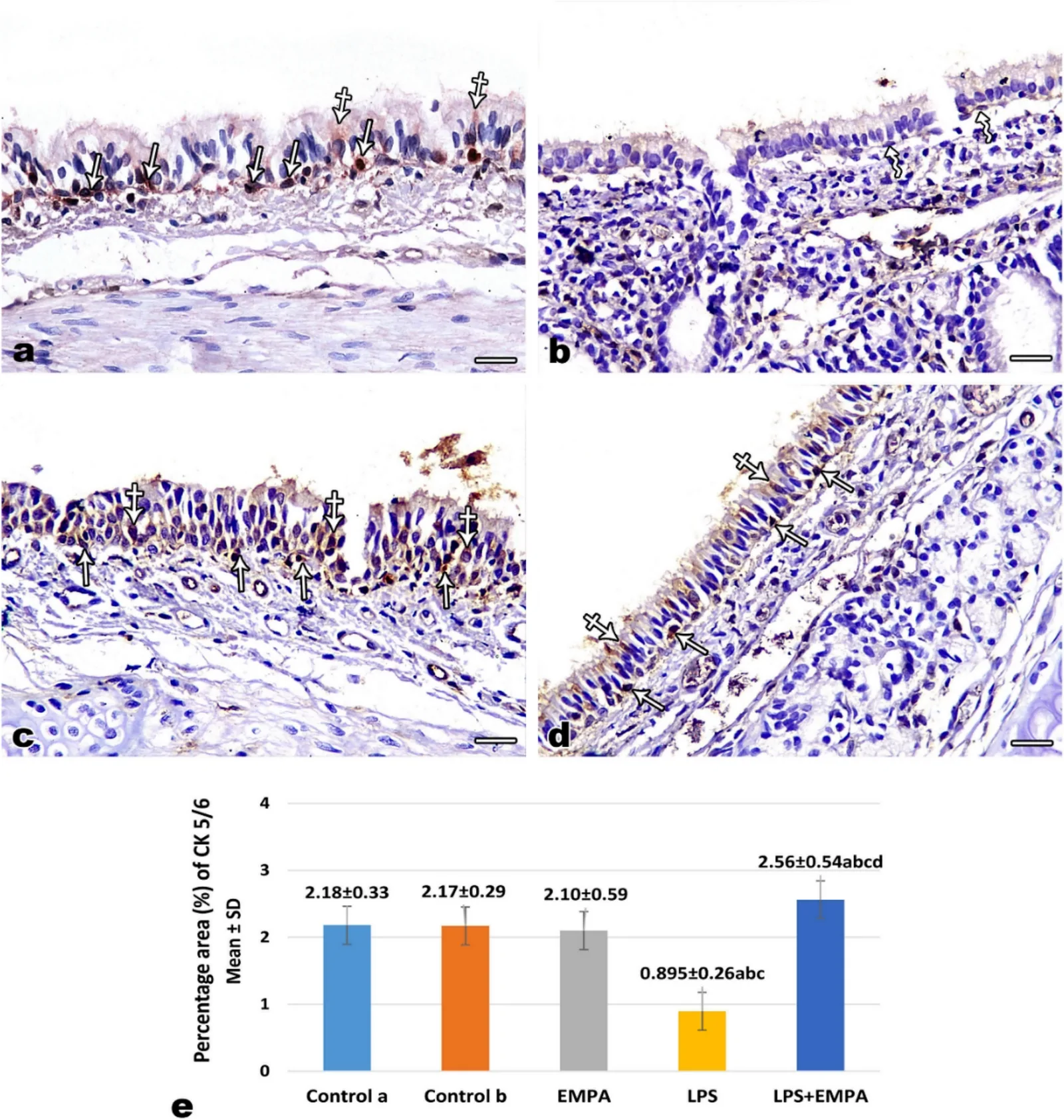
**a**–**d** CK5/6 immunostaining in trachea of the study groups (**a** control group;** b**,** c** LPS group;** d** LPS + EMPA group).** a** showing  strong positive cytoplasmic CK5/6 immunostaining mainly confined to the basal stem cells (arrows). Weak reaction appears in few suprabasal cells (crossed arrows). **b** Apparent loss of CK5/6 expression in the basal stem cells (zigzag arrows) is seen. **c** Positive CK5/6 immunoreactivity is observed in some epithelial areas in both basal (arrows) and suprabasal (crossed arrows) cells. **d** Restoration of CK5/6 expression predominantly in the basal stem cells (arrows) with limited suprabasal (crossed arrows) reactivity (anti-CK5/6 × 400; scale bar = 25 μm). **e** Percentage area (%) of tracheal CK5/6 immunostaining of study groups. One-way ANOVA test is used. Data are expressed as mean ± SD. *P* < 0.05 is significant; a indicates significance against control group a, b significance against control group b, c significance against EMPA group, d significance against LPS group. (*n* = 6 rats/group)

A strong CK5/6 positive cytoplasmic immune expression was specifically localized to the tracheal basal stem cells in the control and EMPA groups. Absent or weak CK5/6 immune expression was found in a few suprabasal cells. There was a marked reduction or loss of CK5/6 immune expression in the basal stem cells in most epithelial areas in the LPS group. However, in some regions there was positive CK5/6 immune expression in the basal stem and suprabasal cells. The LPS + EMPA group showed restoration of CK5/6 immune expression mainly in the basal stem cells with limited suprabasal immune expression (Fig. 8a–d). The percentage area (%) of tracheal CK5/6 was significantly lower (*p* < 0.05) in the LPS group compared to the control and EMPA groups. However, it was significantly higher (*p* < 0.05) in the LPS + EMPA group compared to the control, EMPA, and LPS groups (Fig. 8e).

### EMPA mitigated tracheal ultrastructural alterations in LPS-treated rats

**Fig. 9:**
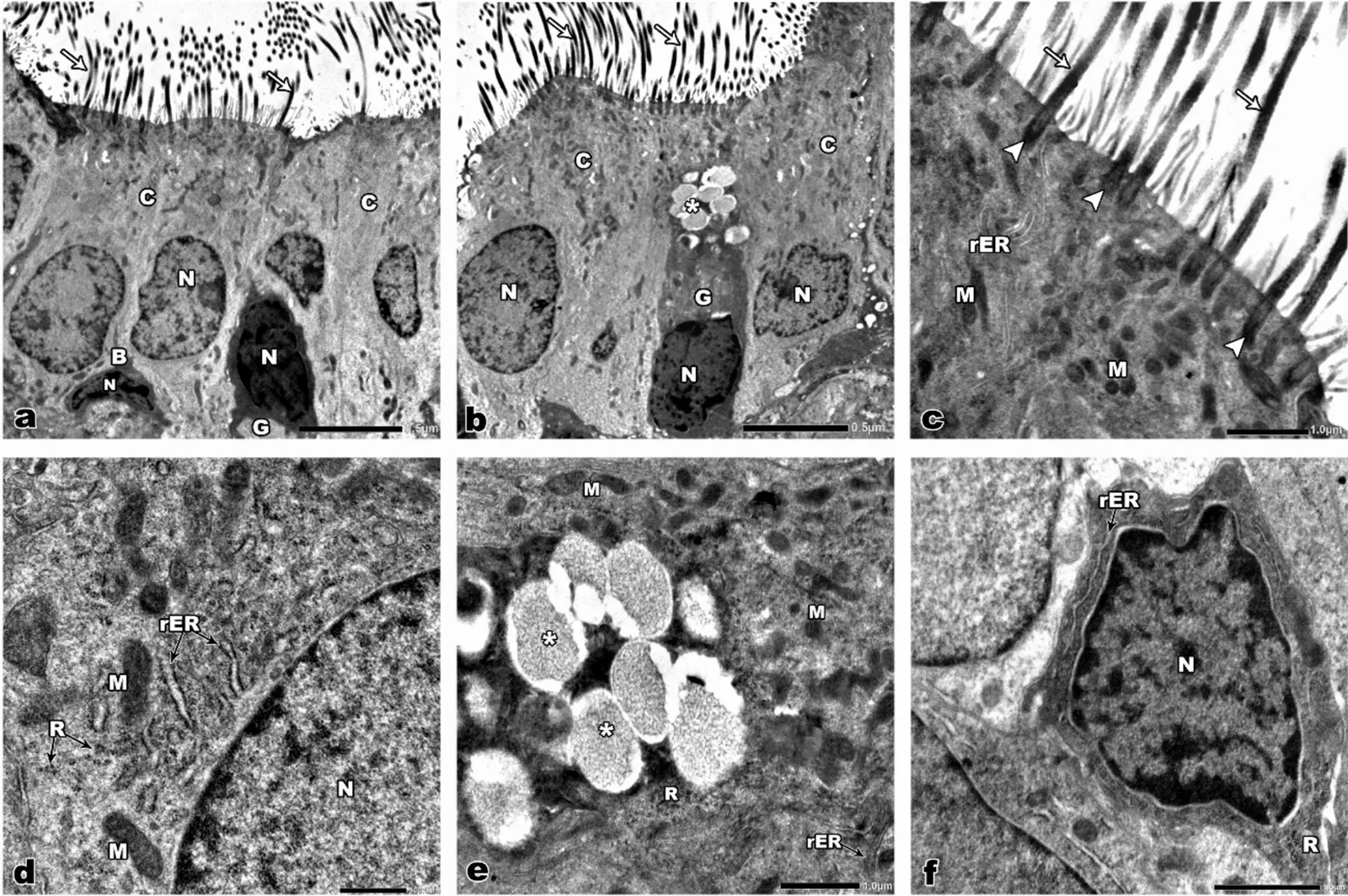
Electron micrographs of the tracheal mucosa of control group. **a**, **b** The tracheal pseudostratified columnar ciliated epithelium reveals the columnar ciliated cells (C), the more electron-dense goblet cells (G), and basal cells (B). The columnar ciliated cells contain rounded to oval euchromatic nuclei (N) with small patches of peripheral heterochromatin. Regularly arranged cilia (arrows) are present on their apical surface. The goblet cells exhibit basally located heterochromatic nuclei (N), and their apical cytoplasm contains large electron-lucent mucin granules (asterisks). The basal cells appear small, triangular, and show heterochromatic nuclei (N). **c** The apical region of the columnar ciliated cells shows cilia (arrows) with numerous basal bodies (arrow heads) anchoring the cilia to the cytoplasm. The cytoplasm contains mitochondria (M), rough endoplasmic reticulum (rER). **d** A ciliated cell shows part of a euchromatic nucleus (N), its cytoplasm contains mitochondria with well-defined cristae (M), rough endoplasmic reticulum (rER), and free ribosomes (R). **e** The apical region of a goblet cell shows large mucin granules of variable electron density (asterisks). The cytoplasm shows numerous mitochondria (M), rough endoplasmic reticulum (rER), and free ribosomes (R). **f** A basal cell shows a heterochromatic nucleus (N); its cytoplasm contains rough endoplasmic reticulum (rER), and free ribosomes (R) (TEM, **a**, **b** ×1500; scale bar = 0.5 µm, **c**, **e** ×6000; scale bar = 1 µm, **d** ×10,000; scale bar = 500 nm, f ×8000; scale bar = 1 µm) (*n* = 6 rats)

Electron microscopic examination of the control and EMPA groups revealed the tracheal pseudostratified columnar ciliated epithelium that comprised columnar ciliated cells, goblet cells, and basal cells. The columnar ciliated cells showed regularly arranged cilia on their apical surfaces with their basal bodies. Their cytoplasm showed euchromatic nuclei with patches of peripheral heterochromatin, mitochondria, rough endoplasmic reticulum (rER) cisternae, and free ribosomes. The goblet cells appeared more dense and showed basally situated heterochromatic nuclei. Their apical parts contained mucous granules of variable electron densities. Mitochondria, rER, and free ribosomes were located in their cytoplasm. The basal cells were small, triangular, and showed heterochromatic nuclei, some rER cisternae, and free ribosomes (Fig. 9).

**Fig. 10:**
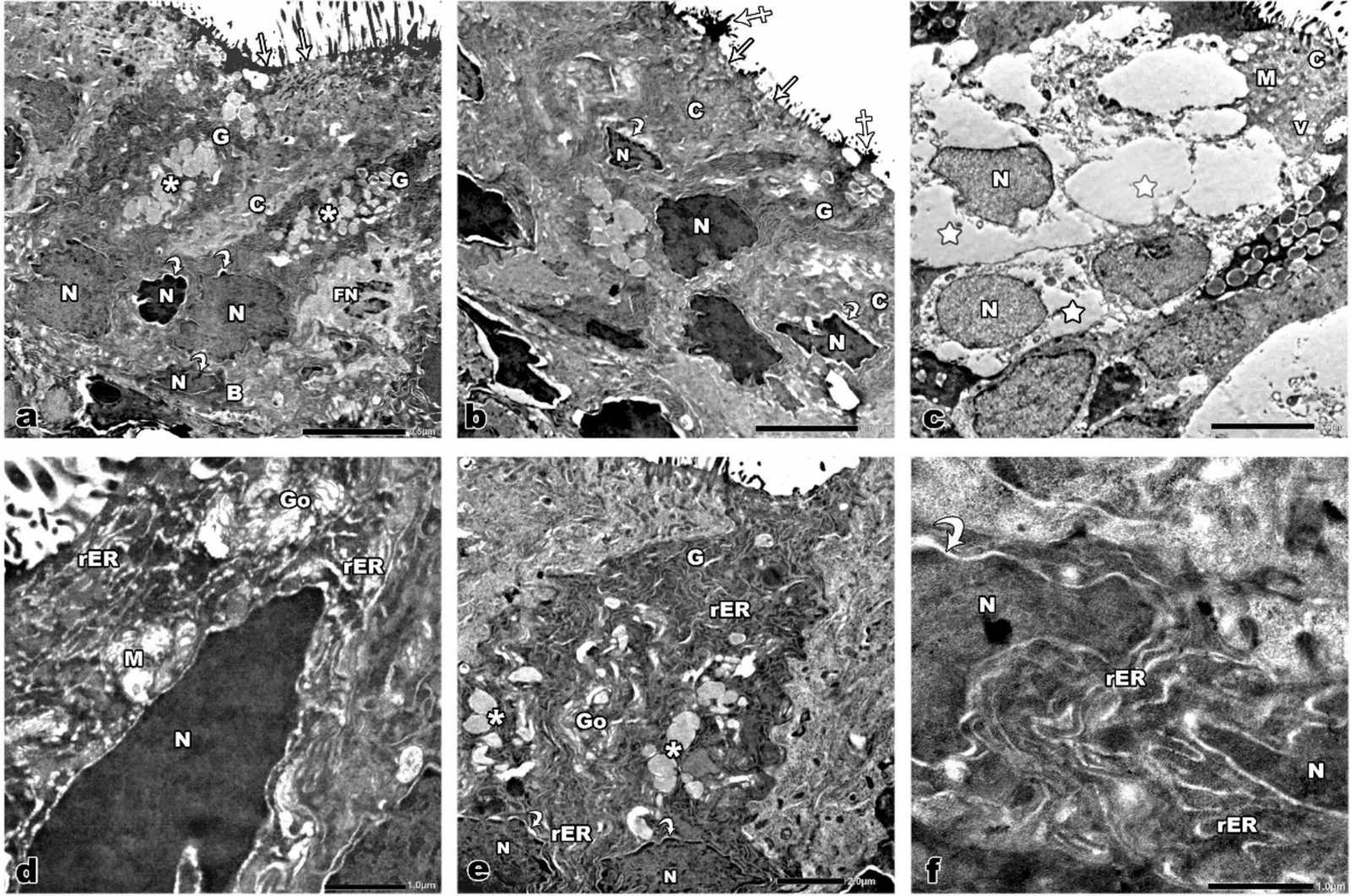
Electron micrographs of the tracheal mucosa of the LPS group. **a**,** b** The ciliated cells (C) show shrunken, irregular, heterochromatic nuclei (N) surrounded by wide perinuclear space (curved arrow), with loss of the apical cilia (arrows). A ciliated cell with a fragmented nucleus (FN) is also observed. Goblet cells (G) contain irregular nuclei (N), wide perinuclear spaces (curved arrows), and display prominent secretory activity; some of them contain numerous, large mucin granules (asterisks), while others show apparent mucin extrusion (crossed arrows). The basal cell shows nucleus (N) with wide perinuclear space (curved arrows). **c** Ciliated cells (C) exhibit vacuoles (V), rarefied cytoplasm (stars), containing residual electron-lucent nuclei (N) and mitochondria with lost cristae (M). **d** Part of a ciliated cell contains an electron-dense nucleus (N); its cytoplasm shows degenerated swollen mitochondria (M) with cristolysis, dilated rough endoplasmic reticulum (rER), and dilated Golgi saccules (Go). **e** Goblet cells (G) show dilated rough endoplasmic reticulum (rER) cisternae, dilated Golgi saccules (Go), and mucin granules (asterisks) with variable electron density. Their nuclei (N) are irregular with wide perinuclear space (curved arrows). **f** The basal cells contain irregular nuclei (N) surrounded by wide perinuclear space (curved arrow); their cytoplasm shows dilated rough endoplasmic reticulum (rER) (TEM, **a**–**c** ×1500; scale bar = 0.5 µm, **d** ×6000; scale bar = 1 µm, **e** ×2500; scale bar = 2 µm, **f** ×8000; scale bar = 1 µm) (*n* = 6 rats)

The LPS group demonstrated evident degenerative ultrastructural alterations. The ciliated cells exhibited loss of their apical cilia. Their nuclei were shrunken irregular and heterochromatic with wide perinuclear spaces. Other nuclei were fragmented. The cytoplasm showed swollen degenerated mitochondria with cristolysis, dilated rER, dilated Golgi and vacuoles. Cytoplasmic rarefaction was also noticed in some ciliated cells. The goblet cells revealed irregular nuclei with wide perinuclear space, dilated rER, and dilated Golgi. The goblet cells showed prominent secretory activity, some of them had numerous mucin granules, and others exhibited mucin extrusion. The basal cells revealed irregular nuclei with widening of the perinuclear space and dilated rER (Fig. 10).

**Fig. 11:**
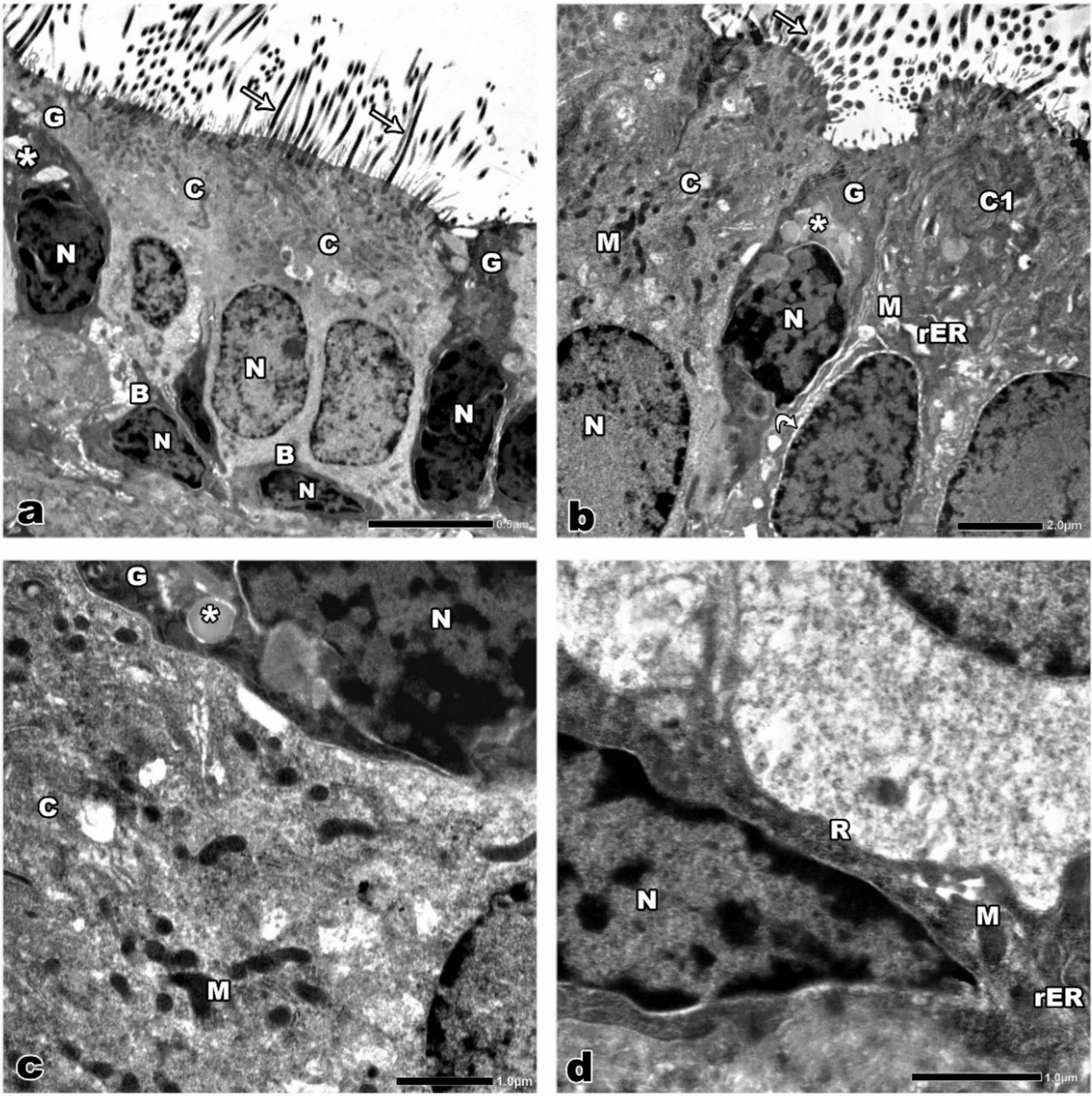
Electron micrographs of the tracheal mucosa of the LPS + EMPA group. **a** The ciliated cells (C) show oval euchromatic nuclei (N), intact mitochondria (M), and regularly arranged cilia (arrows). The goblet cells (G) contain heterochromatic nuclei (N), and the apical cytoplasm contains some mucin granules (asterisks) with variable electron density. The basal cells (B) contain heterochromatic nuclei (N). **b**, **c** (**c** is a higher magnification of **b**): The ciliated (C) cells contain mitochondria with well-defined cristae (M), dilated rough endoplasmic reticulum (rER), and few vacuoles (V). Another ciliated cell (C1) shows some degenerative changes; its cytoplasm contains degenerated mitochondria (M), dilated rough endoplasmic reticulum (rER). Wide perinuclear space is also noticed (curved arrow). The goblet cells (G) show preserved structure with heterochromatic nucleus (N) and mucin granules (asterisks). **d** shows a  basal cell with heterochromatic nucleus (N), mitochondria (M), rough endoplasmic reticulum (rER), and free ribosomes (R). (TEM, **a** ×1500; scale bar = 0.5 µm, **b** ×2500; scale bar = 2 µm, **c** ×6000; scale bar = 1 µm, **d** ×8000; scale bar = 1 µm) (*n* = 6 rats)

On the other hand, the LPS + EMPA group revealed noticeable improvement compared to the LPS group. The tracheal columnar ciliated, goblet, and basal cells appeared with relatively preserved ultrastructure. However, some ciliated cells showed some degenerative changes; they showed wide perinuclear space, degenerated mitochondria, and dilated rER (Fig. 11).

### EMPA downregulated IL-6/JAK/STAT3 signaling pathway in trachea

**Fig. 12:**
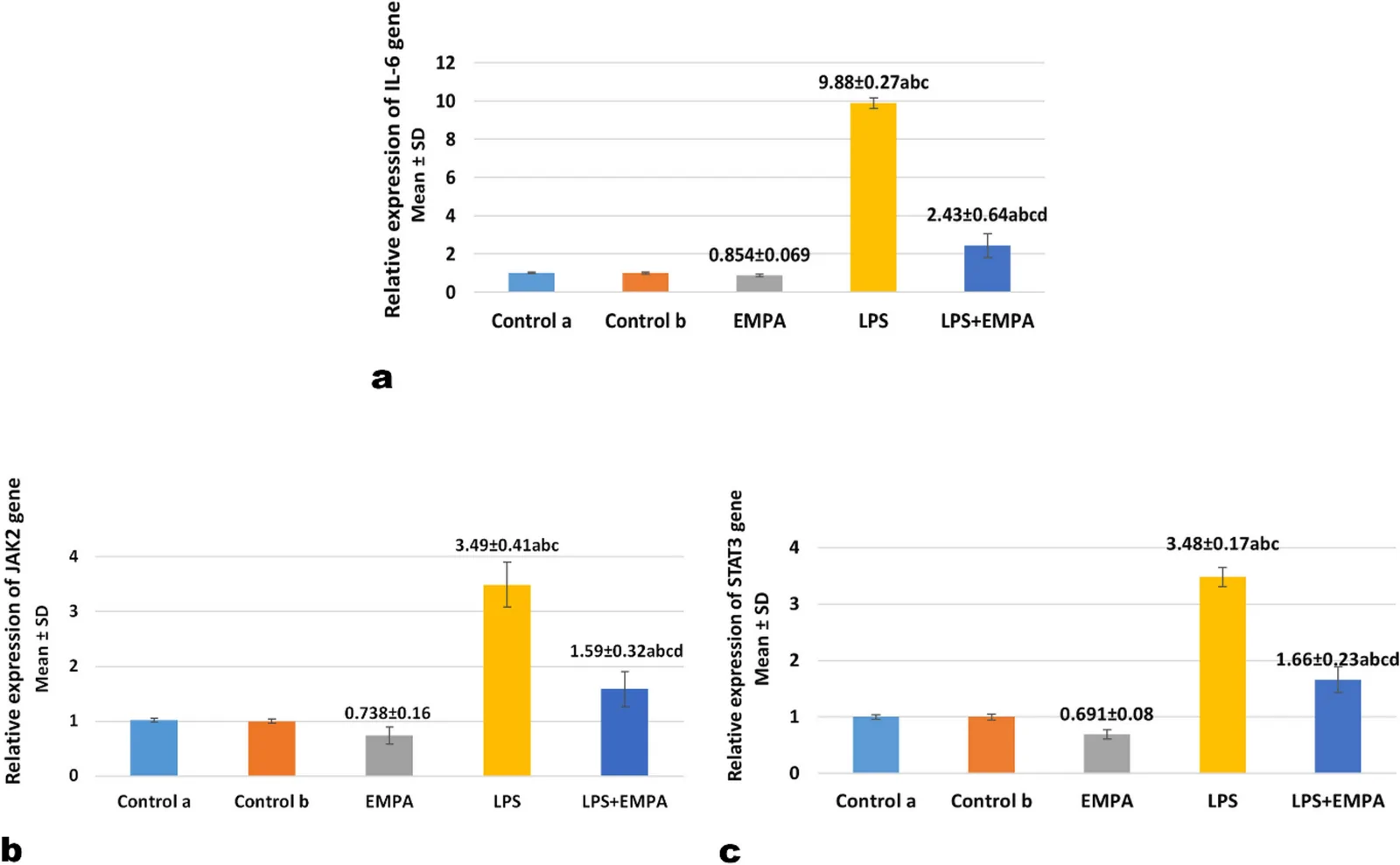
Relative expression of* IL-6* (**a**),* JAK2* (**b**), and* STAT3* (**c**) genes in the different study groups by RT-qPCR. One-way ANOVA test is used. Data are expressed as mean ± SD. *P* < 0.05 is significant; a indicates significance against control group a, b significance against control group b, c significance against EMPA group, d significance against LPS group. (*n* = 6 rats/group)

The LPS group exhibited a marked and significant (*p* < 0.05) upregulation of* IL-6*,* JAK2*, and* STAT3* gene expression compared to both control and EMPA groups. The co-administration of EMPA with LPS in the LPS + EMPA group significantly downregulated (*p* < 0.05) the gene expression of* IL-6*,* JAK2*, and* STAT3* compared to the LPS group, although the expression of the previous genes remained significantly higher (*p* < 0.05) than in the control and EMPA groups (Fig. 12).

## Discussion

The tracheal epithelium represents a critical first line of defense against inhaled pathogens and toxins, and its integrity is important in maintenance of mucociliary clearance and overall airway homeostasis. In this context, tracheal epithelial cells are considered key regulators of airway pathophysiology in various respiratory diseases associated with tracheal injury, including allergic asthma, chronic obstructive pulmonary disease, and cystic fibrosis (Xie et al. 2021). LPS, which is derived from cell wall of Gram-negative bacteria, is commonly used in experimental models to induce airway injury to mimic tracheal pathological condition (Xie et al. 2021; Karaman et al. 2025).

Previous studies have extensively investigated the protective effects of the SGLT2 inhibitor EMPA in various models of organ injury, including chemotherapy-induced cardiotoxicity (Goje et al. 2026) and nephrotoxicity (Tawfik et al. 2026). In addition, accumulating evidence has demonstrated its protective role in respiratory disorders, such as bleomycin-induced pulmonary fibrosis (El-Horany et al. 2023) and airway inflammation and fibrosis associated with asthma (Medeiros et al. 2025). Furthermore, recent findings have highlighted the neuroprotective potential of EMPA against LPS-induced depressive-like behavior (Borikar et al. 2025).

Despite these promising studies, and although EMPA has shown consistent anti-inflammatory and antioxidant effects across multiple organ systems (Raut and Cucullo 2025; Tawfik et al. 2026), the potential role of EMPA in modulating the LPS-induced tracheal injury, particularly through the IL-6/JAK/STAT3 signaling pathway involved in EMT and the effect on tracheal basal stem cells, remains largely unexplored. Accordingly, the current study was designed to investigate the potential protective effects of EMPA against LPS-induced tracheal injury, with particular emphasis on EMT and tracheal basal stem cells.

In the present study, LPS administration revealed marked structural alterations in the tracheal mucosa, including disarrangement and epithelial disruption together with an exaggerated inflammatory response, establishing a model of LPS-induced airway injury. These findings were consistent with a previous study that stated LPS can induce the features of acute inflammation in the tracheal epithelium, facilitating extensive tissue damage (Xie et al. 2021).

The histopathological alterations observed in the tracheal mucosa of the LPS group can be primarily attributed to LPS-induced oxidative stress and inflammation that lead to apoptosis (Karaman et al. 2025). In our study, exposure to LPS resulted in oxidative stress as evidenced by a significant increase in tissue MDA level and a significant reduction of antioxidant enzyme activities, including SOD and CAT, compared to control and EMPA groups. This disparity between the generation of reactive oxygen species (ROS) and the antioxidant mechanisms likely played a role in the observed tracheal damage. These findings were in accordance with previous reports, spotlighting the role of LPS in oxidative stress (Xu et al. 2022; Skibska et al. 2023). ROS damage cellular lipids and proteins, leading to dysfunction of membranes, impaired cellular metabolism, and disrupted mitochondrial membrane integrity. Mitochondrial damage leads to activation of the intrinsic apoptotic pathway (Isaac et al. 2023; Huang et al. 2025). This mechanism may account for the extensive epithelial loss observed in the present study, where some areas appeared nearly completely denuded. Moreover, it might explain the significant increase in the percentage area of Bax immunoreactivity, a pro-apoptotic marker, observed in the LPS-treated group compared to the control and EMPA groups (Kutlay et al. 2026).

These findings were further supported by the marked degenerative ultrastructural alterations of tracheal ciliated cells observed in this study. The shrunken, heterochromatic, and fragmented nuclei, swollen degenerated mitochondria, dilated rER, cytoplasmic rarefaction, and vacuolations confirm the occurrence of severe oxidative stress and apoptosis at subcellular level (Isaac et al. 2023). Furthermore, the ultrastructural observation of the goblet cells prominent secretory activity could also be attributed to LPS-induced oxidative stress and inflammation. The airway response to the insult by increasing* MUC* gene expression, enhancing mucin production and secretion, is considered a protective mechanism (Chen et al. 2025; Akparova et al. 2026; Feng et al. 2026).

Marked inflammatory cell infiltration that was observed in the lamina propria and submucosa of LPS group further supports the role of inflammation in LPS-induced tracheal injury. The relationship between oxidative stress and inflammation is a vicious cycle of persistent tissue injury and disease progression. High ROS level enhances pro-inflammatory signaling pathways and promotes more tissue damage. On the contrary, inflammation enhances ROS production through immune cell activation and redox-sensitive pathways (Manful et al. 2025). In line with these findings, LPS treatment in the current study resulted in a significant increase in the serum levels of pro-inflammatory cytokines, including IL-6, IL-1β, and TNF-α, compared to the control and EMPA groups, creating an inflammatory tracheal injury model. These findings are consistent with Liu et al. (2018) in the epithelial cells of human airway and could be attributed to induction of airway inflammation through LPS-induced TLR4 activation, which results in downstream NF-κB signaling and subsequent release of pro-inflammatory cytokines (Wiger et al. 2025).

In addition, the observed loss and disorganization of cilia that were evident in both H&E-stained sections and ultrastructural examinations might be attributed to the combined effects of inflammatory cytokines and oxidative stress. Pro-inflammatory cytokines have been shown to impair ciliogenesis and disrupt differentiation of the ciliated cells, while oxidative stress and ROS can damage the microtubule-based axonemal structure of cilia, leading to ciliary dysfunction or complete loss (Moruzzi et al. 2022; Murphree-Terry et al. 2025). These findings are consistent with recent reports demonstrating that LPS induces ciliary dysfunction and loss of the normal ciliated epithelial phenotype (Yang et al. 2025a).

In the present study, the presence of a single layer of cuboidal epithelium in the LPS group might represent an early regenerative response following epithelial injury. Alternatively, it might reflect a dedifferentiated state of the damaged epithelium, in which cells lose their specialized ciliated phenotype as a consequence of oxidative stress and inflammatory insults (Isaac et al. 2023). In addition, LPS-induced inflammation has been reported to disrupt vitamin A homeostasis with subsequent hyporetinolemia (Rubin et al. 2017). Vitamin A is important for the maintenance of normal epithelial differentiation through regulation of retinoic acid-mediated genes. Therefore, its deficiency has been associated with epithelial metaplasia in the respiratory tract, where the normal pseudostratified ciliated epithelium may be replaced by simple cuboidal or even squamous epithelium (Sapmaz et al. 2017; Zaragosi et al. 2026). This mechanism may further explain the altered epithelial morphology observed in the present study. Furthermore, the observed epithelial hyperplasia and structural irregularity in a few regions in the LPS group were consistent with previous studies reporting that LPS induces irregularity, hyperplasia, and increase in the thickness of tracheal epithelium (Grayson et al. 2025). This might reflect a disorganized or defective post-injury regenerative response in which the epithelial cells lost their normal characteristics and junctions between them (Chen et al. 2024).

The vascular congestion detected in the current study might be explained by the inflammatory response triggered by LPS, leading to endothelial injury, vasodilation, and increased vascular permeability (Bahashwan et al. 2025).

In contrast, the LPS + EMPA group demonstrated a remarkable improvement in tracheal mucosal architecture with preservation of the epithelial lining and cilia that was detected by light and electron microscopic examination. Moreover, morphometric analysis revealed a significant elevation in the epithelial thickness compared to the LPS group, restoring it to values that were not significantly different from those of the control group. This improvement was accompanied by a significant reduction in the percentage area of Bax immunostaining compared with the LPS group. These findings were accompanied by decreased MDA levels, restoration of SOD and CAT activities, and reduced serum levels of proinflammatory cytokines, indicating attenuation of oxidative stress and inflammation (Yaribeygi et al. 2023; Magadmi et al. 2025). EMPA has been reported to reduce ROS production and enhance cellular redox balance, thereby limiting oxidative damage to cellular membranes and proteins. Furthermore, it enhances mitochondrial efficiency by reducing ROS production and activating AMP-activated protein kinase (AMPK) and nuclear factor erythroid 2-related factor 2 (Nrf2) pathways, which stimulate the production of antioxidant enzymes (Borikar et al. 2025). In addition, EMPA suppresses inflammatory signaling pathways, including TLR4/NF-κB activation, reducing the production of pro-inflammatory cytokines and modulating immune cell activity (Borikar et al. 2025; Rykova et al. 2025; Cliff et al. 2026). These findings were consistent with previous studies showing that EMPA exerts strong antioxidant and anti-inflammatory effects in acute septic renal injury (Maayah et al. 2021) and in cardiac inflammation of non-diabetic models of heart failure (Koyani et al. 2020). In addition, EMPA showed an antiapoptotic effect in hepatotoxicity models (Asgari and Kalhori 2025).

One of the major findings in our study after LPS administration is the significant upregulation of* IL-6* and its downstream mediators,* JAK2* and* STAT3* gene expression by RT-qPCR in tracheal tissues compared to the control and EMPA groups. It was confirmed that the IL-6/JAK/STAT3 pathway is essential in driving chronic inflammation and tissue scarring. Phosphorylated STAT3 translocates to the nucleus and promotes transcription of inflammatory, fibrotic and EMT-related genes. So, the observed activation of this pathway confirms its critical role in LPS-induced tracheal injury (Qin et al. 2020; Tang et al. 2025).

The LPS group showed a significant decrease in percentage area of E-cadherin immunostaining and a significant increase in percentage area of vimentin compared to the control and EMPA groups. In addition, there was a significant increase in the percentage area of collagenous fibers in the lamina propria and submucosa in Masson trichrome-stained sections. These results could be attributed to LPS-induced EMT and fibrosis (Qin et al. 2020). These results were in agreement with previous studies reporting that LPS induces EMT in alveolar epithelial cells and promotes lung fibrosis (Qin et al. 2020; Yang et al. 2022). On the other hand, the LPS + EMPA group showed downregulation of* IL-6*,* JAK2*, and* STAT3* expression, significant increase in percentage area of E-cadherin, and significant decrease in percentage area vimentin and collagenous fibers compared to the LPS group. EMPA has been shown to modulate the IL-6/JAK/STAT3 signaling pathway (Zhou et al. 2024), interfere with EMT (Yang et al. 2025b), and have marked anti-fibrotic activity (El-Horany et al. 2023; Rolski and Mączewski 2025) as it inhibits fibroblasts with subsequent decrease in collagen deposition (Rolski and Mączewski 2025).

E-cadherin, a key epithelial junction protein, is important for preserving epithelial integrity and tissue homoeostasis. In contrast, vimentin, a mesenchymal marker, induces cell migration during EMT by formation of cell processes and reduction of cell adhesion (Li et al. 2026). During EMT, the transitioning epithelial cells gain migratory potential that enables them to invade the epithelial basement membrane into the underlying lamina propria and submucosa and differentiate into fibroblasts, leading to increase in extracellular matrix and collagen deposition (Dey et al. 2025).

Prior studies have demonstrated that basal cells of the tracheobronchial epithelium constitute a multipotent progenitor cell population capable of self-renewal and differentiation after injury (Murthy et al. 2025). A previous study stated that repair of tracheal epithelium involves migration, proliferation, and differentiation of basal cells to restore epithelial integrity. The basal tracheal stem cells expressing CK5/6 show changes in size, shape, and position during the repair process. It changes from thin cells covering the basement membrane to larger cells with enlarged nuclei in the hyperplastic regenerative epithelium and then returns into small pyramidal cells (Ruysseveldt et al. 2021).

The current study showed large area of denuded epithelium or monolayer organization in the LPS group, indicating loss of the basal stem cells. However, in some regions there was a positive CK5/6 immunoexpression in the basal stem and suprabasal cells which represents defective regenerative ability after injury. In parallel, there was a significant reduction in percentage of CK5/6 immunostaining. These findings were further supported by the ultrastructural degenerative observations in the basal cells, which showed shrunken, irregular nucleus and dilated rER, confirming the cellular stress and defective regenerative capacity (Isaac et al. 2023).

On the other hand, the LPS + EMPA group showed a significant increase in percentage area of CK5/6 immune expression, suggesting restoration of the basal stem cells. Although there is currently no evidence of a direct stimulatory effect of EMPA on tracheal basal stem cells, our results suggested that the antioxidant and anti-inflammatory effect of EMPA create a supportive healthy environment that preserves the tracheal basal stem cells. The current results align with recent studies that illustrate the role of airway basal stem cells in epithelial regeneration after injury (Liu et al. 2025a; Zhao et al. 2025).

## Conclusion

Empagliflozin offers substantial protection against LPS-induced tracheal injury, primarily through its antioxidant and anti-inflammatory properties. In addition, it alleviates EMT, attenuates fibrotic changes together with enhanced CK5/6 expression, suggesting preservation of the tracheal basal stem cell population. Further research is required to investigate additional molecular mechanisms for the protective effects of EMPA against tracheal injury. These findings provide a foundation for future studies exploring the therapeutic potential of EMPA in inflammatory airway diseases. However, further preclinical and clinical studies are needed to confirm its efficacy and safety before translation into clinical practice.

## Limitations and recommendations

The current study provided valuable information about the effectiveness of EMPA in protecting against LPS-induced tracheal injury; however, it has certain limitations. First, the IL-6/JAK/STAT3 pathway was assessed only at the mRNA level, while protein expression and phosphorylation status, particularly p-STAT3, were not evaluated. Therefore, additional studies incorporating protein-based analyses are needed to further confirm the involvement of this pathway in the protective effects of EMPA. Second, although CK5/6 is a recognized marker of airway basal stem/progenitor cells, assessment of additional stem cell and proliferation markers, such as p63 or Ki-67, would provide a more comprehensive evaluation of stem cell function and regenerative capacity. Therefore, further studies are warranted to better characterize the regenerative potential of the basal stem cell population.

## Data Availability

No datasets were generated or analysed during the current study.
